# P-1075. Distribution of Community Acquired Multidrug Resistant Bacterial Infections and Association with Proximity to Healthcare Facilities in Cobb County, GA

**DOI:** 10.1093/ofid/ofaf695.1270

**Published:** 2026-01-11

**Authors:** Alexandra Kershteyn, Margaret Omatsone

**Affiliations:** Wellstar Kennestone Hospital, Marietta, GA; Wellstar Kennestone Hospital, Marietta, GA

## Abstract

**Background:**

In the United States, community-acquired Extended Spectrum Beta-lactamase (ESBL) infections are increasing. Risk factors for acquisition remain limited. Municipal water systems, access to antibiotic prescription and sanitary conditions may facilitate transmission. This study examines the relationship between residential proximity to healthcare facilities and risk of infection.

**Figure:**
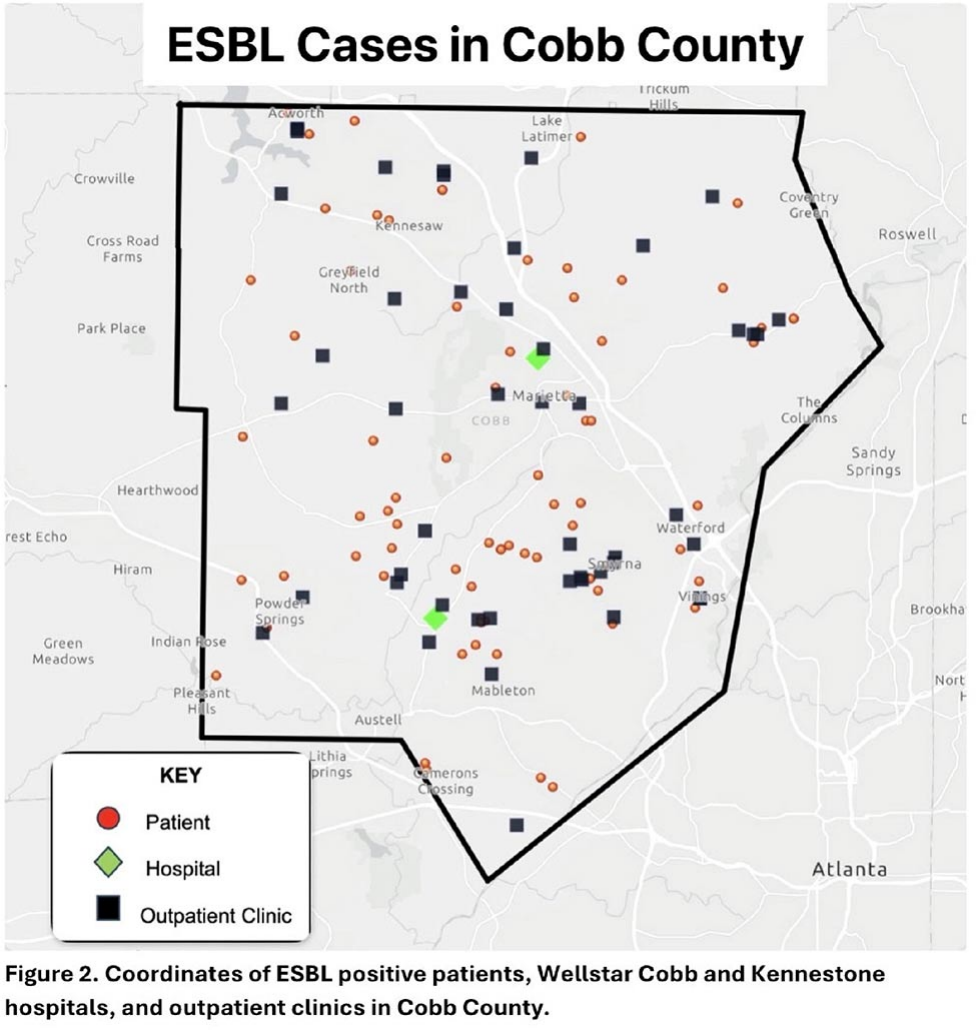

**Methods:**

We conducted a 24-month retrospective study of Cobb County residents with ESBL-positive cultures from Wellstar laboratories. Exclusion criteria included hospitalization in the past 3 months, residing in a nursing facility, discharge from a rehabilitation facility within the past 6 months, culture taken ≥ 3 days after admission, antibiotic use within the past 3 months, and prior ESBL infection. Poisson regression analyzed the distance from patients' homes to the nearest clinic and hospital to create a prediction model for incidence of ESBL infections. Geospatial information systems (GIS) mapped patient homes and healthcare facilities to visualize distribution patterns.

**Figure:**
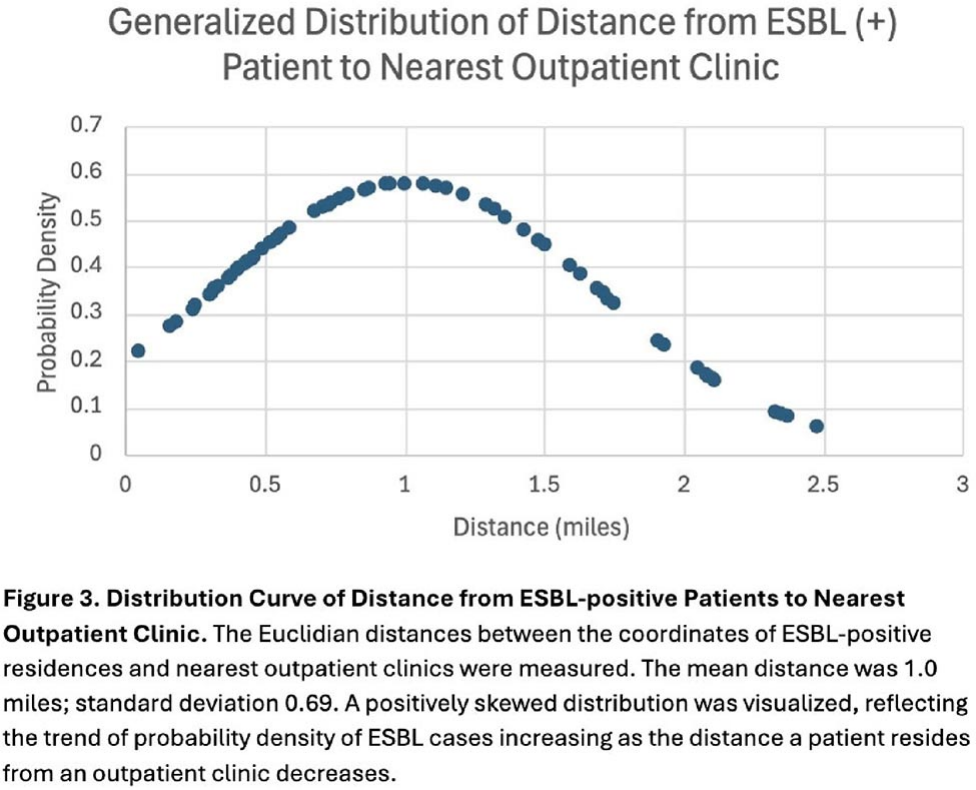

**Results:**

Among 3052 ESBL-positive cultures in 2023–2024, 73 patients met study criteria (mean age 64 years; 66.7% female). Isolates included Escherichia coli (83.5%), Klebsiella pneumoniae (11.4%), Enterobacter cloacae (3.8%), and Proteus species (1.3%) from urine (83.1%), blood (9.6%), wounds (4.8%), and sputum (2.4%). Nitrofurantoin was the most frequently prescribed outpatient empiric antibiotic (36.4%). Poisson regression revealed a 60.5% reduction in ESBL infection frequency for every 1 mile away from an outpatient facility (95% CI, 0.439–0.832; p = 0.002) and a non-significant 92.6% reduction per mile from hospitals (95% CI, 0.857–1.0; p = 0.05).

**Conclusion:**

A higher frequency of first-time ESBL infections was observed among patients living closer to outpatient healthcare facilities, with a 60% decrease in infections per mile of increased distance. No significant trend was noted with hospitals. Future studies may incorporate patient demographics, antibiotic prescription patterns, community sanitary conditions and advanced GIS tools to refine predictive models and identify transmission clusters for targeted public health interventions.

**Disclosures:**

All Authors: No reported disclosures

